# Multi-level community dissemination of public health research: insights from the fuel to pot project case study in Kenya and Malawi

**DOI:** 10.3389/fpubh.2026.1702368

**Published:** 2026-05-28

**Authors:** Limbani Kalumbi, Heather Price, Line Caes, Fred Orina, Tracy Chasima, Lusizi Kambalame, Moses V. M. Chamba, Sian E. Lucas, Hellen Meme, Sean Semple, Isabelle Uny

**Affiliations:** 1 Public and Environmental Health Sciences Department, Malawi University of Business and Applied Sciences, Blantyre, Malawi; 2 Department of Biological and Environmental Sciences, University of Stirling, Stirling, United Kingdom; 3 Department of Psychology, Faculty of Natural Sciences, University of Stirling, Stirling, United Kingdom; 4 Centre for Respiratory Diseases Research, Kenya Medical Research Institute, Nairobi, Kenya; 5 Department of Language and Communication Studies, Malawi University of Business and Applied Sciences, Blantyre, Malawi; 6 Department of Physics and Biochemical Sciences, Malawi University of Business and Applied Sciences, Blantyre, Malawi; 7 Department of Social Work, University of Stirling, Stirling, United Kingdom; 8 Faculty of Health Sciences and Sport, Institute for Social Marketing and Health, University of Stirling, Stirling, United Kingdom

**Keywords:** community engagement, household air pollution (HAP), interactive dissemination, participatory methods, research dissemination

## Abstract

Household air pollution (HAP) from solid fuel use affects nearly 3.6 billion people globally, causing 3.2 million deaths annually. Despite extensive research, interventions show limited health benefits, highlighting the need for community-centered approaches that meaningfully engage affected populations in research dissemination and solution co-design. We present a community case study from the Fuel to Pot project (2020–2023), an interdisciplinary study conducted in the informal settlements of Mukuru, Kenya, and Ndirande, Malawi. Following 2 years of participatory research using walking interviews and photovoice, we implemented a cascaded, interactive dissemination model involving sequential community engagement events followed by policymaker discussions. Community dissemination events engaged diverse community members in each location through mobile poster exhibitions featuring local language translations, photovoice images, and simplified data visualizations. Interactive discussions generated contextually relevant recommendations addressing immediate community needs and systemic policy changes. Subsequent policymaker events with key stakeholders further facilitated dialog between community priorities and institutional capacity, strengthening researcher-community-policy relationships. We suggest six principles for community engagement in research dissemination (Engagement, Networks, Accessibility, Capacity strengthening, Trust, and Budgeting) that aim to transform research dissemination from a performative, extractive practice into a collaborative action. This approach enhances the sustainability of interventions, builds local capacity, and addresses ethical imperatives in global and public health research. The principles provide a replicable framework for researchers seeking to move beyond traditional academic dissemination toward more genuine community engagement and the co-creation of effective health interventions.

## 1 Introduction

Recent literature highlights severe challenges in urban air quality across Africa (1–6). In African cities, both ambient air pollution (AAP) and household air pollution (HAP) contribute to elevated concentrations of fine particulate matter (PM2.5), with significant health impacts (1, 7). Yet, the use of solid fuels, such as wood or charcoal, for cooking and heating in the home remains common, with almost 3.6 billion people −47% of the world’s population– still exposed to HAP and suffering other major safety and health risks linked to the use of solid fuels (2). This is leading to a heavy burden of disease with approximately 3.2 million deaths per year from illnesses attributable to HAP and an estimated 91.5 million disability-adjusted life years globally (8). HAP is linked to non-communicable diseases such as stroke, ischemic heart disease, chronic obstructive pulmonary disease, and lung cancer (9–11). An estimated 86% of global human exposure to air pollution occurs in homes in low- and middle-income countries [LMIC] (12). Women and children are the most exposed to HAP (13, 14) due to their roles around cooking. HAP alone causes approximately 700,000 deaths per annum in Africa (1, 3). It is estimated that in 2025, over 1 billion people in sub-Saharan Africa (SSA) relied on solid and polluting fuels for cooking and heating (15). This body of evidence establishes the continued relevance of HAP as a public health concern and provides the contextual basis for examining how affected populations and stakeholders communicate and interpret research findings.

Studies have shown that populations in informal settlements are particularly vulnerable to the harms associated with HAP (14, 16–20). The issue of solid fuel use and related HAP and health harms in informal settlements remains under-researched and is complicated by several interlinked economic, social, and cultural determinants (17, 21). Strategies to reduce HAP exposure in both urban and rural environments have so far focused primarily on technical solutions, such as the introduction of improved cookstoves, cleaner fuels for cooking and heating, or improved ventilation (22–27). Recent systematic reviews of HAP interventions have highlighted that the health benefits for affected populations remain limited, except for small reductions in exposure (28–30). This limitation has shifted attention toward approaches that recognize behavioral, social, and contextual determinants of fuel use and exposure. Based on this, some reviews have therefore called for more community-based (23) and holistic approaches (31), and for an increased understanding of local communities’ preferences regarding fuel use (32, 33). A previous scoping review by this team found limited research on contextualized perceptions of HAP-related health harms and highlighted the need to place those most affected at the centre of intervention design (34). The need to be mindful of cultural and social contexts has also been highlighted (28). This process is key to the co-developing of more contextually appropriate solutions to support the most vulnerable populations as they transition to using cleaner fuels. To accomplish this, more sustained and meaningful engagement with the affected communities in informal settlements is required to ensure alignment with local priorities (35–38). These insights also extend to the manner in which research findings are shared and discussed with those communities and how their views can be communicated to decision-makers.

In the last decade, community engagement in global public health has evolved from traditional consultative frameworks to more participatory, co-creative and empowerment models (39, 40). It now centers on collaborative partnerships, respecting local knowledge and ensuring equity, trust, and sustainability in health interventions, although the impact of community engagement processes is still often under-evaluated (37, 39–42). Brunton et al., in their review, present several models of community engagement in health research: those where community members are central agents in intervention execution; those where partnerships between communities and professionals exist with varying degrees of shared decision-making; and empowerment-based approaches embedded in long-term community development, where communities have further control over interventions and decisions affecting their health (40). This is also described as shared power in social action by Gaventa (43), in his ‘power cube’ model, which describes the various degrees of participation and engagement by different agents with power over others. In this paper, we conceptualize communities not only as geographical or ethnic communities but also as communities of practice. This means, for instance, that we view policymakers and decision makers as a distinct community (one with the power to enact change), due to their common characteristics and interests. This broader conceptualization of “community” underpins our study’s objective of examining dissemination mechanisms across multiple stakeholder groups.

Within the process of community engagement, one recommendation which can support the uptake and design of interventions is the direct dissemination of research findings with the communities most affected (38, 39, 44), as well as those with decision making power. This plays a significant role in ensuring that findings become accessible and actionable while remaining community-informed and checked (45, 46). Dissemination—or the lack thereof—can significantly impact how effectively evidence-based interventions (EBIs) are communicated, understood, and adopted by community stakeholders (44). This process is also essential for avoiding ‘helicopter’ research, the type of extractive research where Global North researchers conduct research in Global South countries without adequate involvement or accountability to local communities (47, 48). More meaningful and sustained involvement is more likely to ensure the sustainability of interventions (49). Further, adequate priority setting is considered to go some way toward ascertaining that the voices of those most affected are heard and represented (50–52).

Recent discourse on responsible research dissemination practices emphasizes the ethical and practical importance of sharing research findings beyond academic circles, particularly with study participants and their communities (45, 46). Literature stresses that responsible dissemination must be timely, transparent, and accessible to foster trust (53, 54). Dissemination is increasingly viewed as a dialogic process that supports the co-creation of effective solutions (55, 56). Building on this foundation, Cunningham-Erves et al. developed and evaluated a structured training module to address the persistent gap in researcher preparedness for community dissemination (57); their work identifies several systemic barriers to effective dissemination, including a lack of institutional support, limited incentives, and misconceptions about community interest, which prevent researchers from prioritizing dissemination. In HAP research, community engagement and inclusion processes are still poorly reported (58), with some notable exceptions (17, 32, 33, 59, 60). In public health research generally, dissemination often still consists merely of publishing findings (61), at times with brief stakeholder briefings or lay outputs shared on social media or presented at community workshops. Still, community access to research outputs is often minimal (37, 42). This gap in practice highlights the need to better understand how dissemination can be conducted in participatory and context-sensitive ways, an objective addressed in this study.

Our own research relating to this paper shows that across informal and urban settlements, residents actively interpret risks, make context-dependent fuel decisions, and employ their own mitigation strategies, although knowledge gaps may persist (60, 62). Participatory approaches such as photovoice further demonstrate that community members can articulate harms and identify locally relevant priorities and potential solutions in their own words (60).

It is in this context that we present a model of community-engaged, interactive dissemination developed within the Fuel to Pot (F2P) project, an interdisciplinary study exploring the use of solid fuels in informal settlements in Kenya and Malawi. This project aimed to understand the issues from the perspective of those most affected and to support the design of sustainable, health-promoting energy transitions for residents of informal settlements. F2P used participatory methodologies: walking interviews [where the researchers conducted the interview with participants as they walked to procure their fuels and returned home and cooked (62–64)], and photovoice [where we trained participants in the use of phone cameras to take photos representing the issues of concern to them in their communities, and to analyze those photos with researchers (60, 65, 66)]. Results from the study have been published elsewhere (60, 62). In this paper, we examine how we extended the participatory aspect to an interactive dissemination of the findings with informal settlement residents and policymakers; we demonstrate how this process can support ethical accountability and epistemic justice in global public health research (67) and provide a practical foundation for the co-production of contextually appropriate intervention ideas. We also propose six guiding principles to support researchers in planning and resourcing community-engaged dissemination across different community types and fields of global public health research engagement processes.

## 2 Fuel to pot study setting and communities

The Fuel to Pot (F2P) project was a two-year study which placed the communities most affected by HAP and solid fuel use for cooking in informal settlements at the heart of the research. Conducted in two informal settlements in Kenya (Mukuru) and Malawi (Ndirande), the research was co-designed with community members to ensure that local experiences, priorities, and knowledge shaped both the study approach and its relevance (60, 62). Mukuru is a large area composed of 30 separate villages located around Nairobi city and is home to an estimated population of 300,000–700,000 people, with most on low income, working in surrounding businesses or industries (17, 68, 69). The wide population estimate for Mukuru reflects the dynamic and transient nature of the population, with frequent in- and out-migration, making accurate enumeration difficult in this informal settlement (70). The population of Mukuru is estimated through community-led enumeration (counting) and detailed mapping, usually, rather than relying solely on official government censuses. Our study took place in the area called Mukuru Kwa Njenga. In Mukuru, housing is informal (made of corrugated iron sheets and other recycled wood or materials) and often lacks adequate sanitation, water and waste disposal systems. Residents use a combination of clean and solid fuels (e.g., LPG and charcoal or wood) for their daily cooking and heating needs (17). The Ndirande Township in Malawi is one of the largest informal settlements in Blantyre, a large city in the South of the country. It is a densely populated area with 97,839 people (71). The settlement lacks formal road networks, clean water systems, drainage or waste disposal systems. In Ndirande, houses are basic (made of locally sourced mud bricks, with some thatched roofs from local vegetation), and many people are on low incomes, working in businesses or markets around the city. In Ndirande, access to clean fuels, particularly electricity, remains low (72, 73), and people use a variety of solid fuels (particularly wood and charcoal). Our study took place in the area called Makata.

## 3 Description of our program of dissemination and direct engagement around the research findings

In this participatory research study, we aimed to foster direct engagement and discussion of the results among those most affected and least heard in informal settlements. To do so, we conducted two interactive visualization and discussion-based dissemination events in Ndirande and Mukuru, the sites where the research had been conducted for 2 years. The process of our dissemination revolved around two ‘cascaded’ events in each location, targeting two key communities: first, the informal settlements community, and second, policymakers and other key stakeholders. The idea was to engage with the informal settlement community first in discussions about the results and then take those points to the policymaker community to gain their perspectives and feedback. Separate meetings were planned and implemented for the community and policymakers to minimize power imbalances and encourage open participation, particularly among participants, while also reflecting logistical and resource constraints that precluded convening a joint session. We did so in both Kenya and Malawi in February 2023.

### 3.1 Community engagement and interactive dissemination in the informal settlement communities

**Figure 1:**
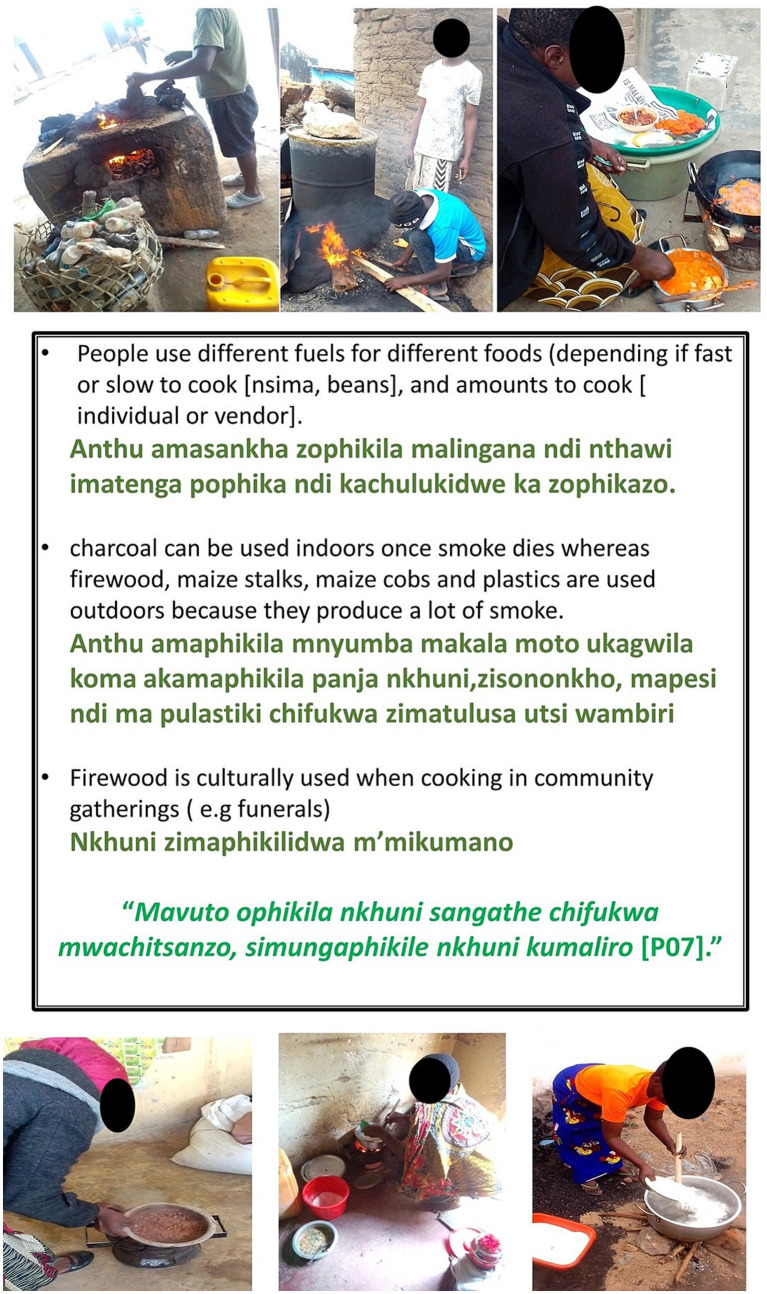
Visualisation poster-community event in Ndirande (Malawi).

To ensure the success of these visualization and discussion events at the informal settlement community level, we relied on the relationships we had built with residents in Mukuru and Ndirande during the F2P study (60, 74). Those who had participated in the research had since self-constituted a small local group that spread the word in advance that our Team was going to hold a visualization and discussion of their research in Mukuru and Ndirande; community leaders also liaized with the research team. The events took place at locations chosen by the informal settlement community members themselves (local community halls). Each event was attended by approximately 50 participants, representing diverse groups of younger and older men and women, local businesspeople, chiefs and village leaders, as well as some local ward councilors. At the start of each event, the local researchers sought collective verbal consent to take notes of points discussed, as well as photos and videos of the proceedings, for use in future publications and on social media. The attendees granted permissions. The discussions took place over a shortened day, with a lunch and refreshments provided. To visualize the results for each informal settlement community in Mukuru and Ndirande, we opted to use a mobile poster exhibition. This not only enabled the wider community to access firsthand the data generated by their fellow residents involved in the research but also expanded access for those with lower literacy levels. Results were visualized on nine large, durable vinyl posters (1.5 × 2.5 m) designed and printed locally, which displayed the F2P data and findings. The posters featured photovoice and walking-interview images, simplified air-pollution graphs, and quotes and text in both English and the local languages (Swahili and Chichewa) to increase accessibility. All the pictures used on the posters had been approved for sharing by the study participants themselves before printing and before each event (one of the posters used is shown in Figure 1). The poster in Figure 1 is an example of a visual used during dissemination to illustrate how cooking practices vary across households and community settings, including differences in fuel choice, cooking location, and food type. It was used during dissemination activities to support dialog with participants.

**Figure 2:**
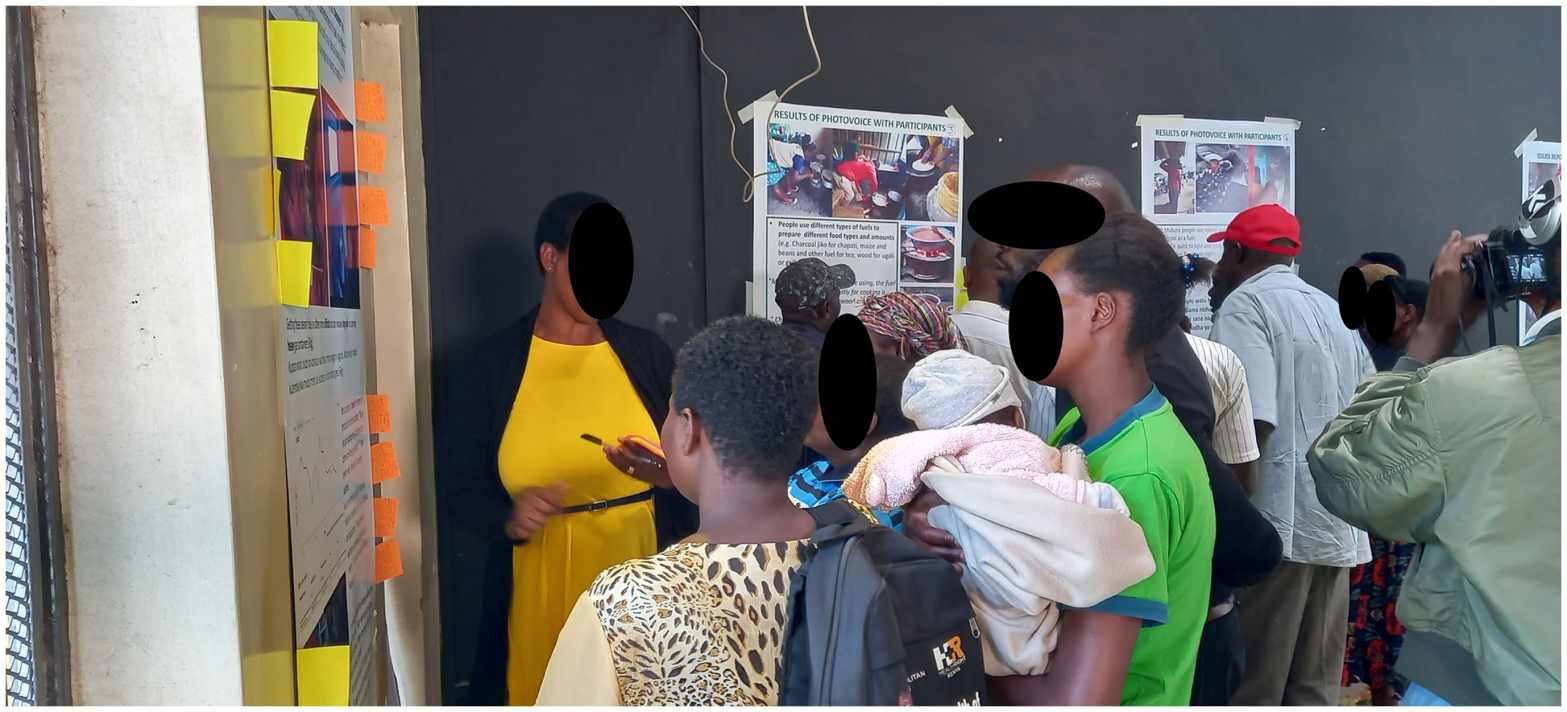
Visualisation discussion with participants in Mukuru (Kenya).

Participants were first given ample time to engage with the posters, walk around the exhibition, consider the visualized results, and ask questions. They were invited to add their own reflections on the results shown using ‘post-it’ notes (on which the researchers took verbatim notes of what was said and which were affixed to each poster). To support broader participation, those who required it had the poster’s text read out to them in their language. At each poster, we placed members of the research team and early-career researchers from the Malawi University of Business and Applied Sciences (MUBAS) and the Kenya Medical Research Institute (KEMRI), all fully briefed in advance and fluent in the local languages, to facilitate discussions, explain the study findings, and respond to participants’ questions. In that way, any resident’s query on the research could be answered directly in their own language. Figure 2 shows a visualization discussion session conducted with participants as part of the dissemination process. To strengthen our research capacity as an interdisciplinary, multi-country team, the full research teams from Kenya and Malawi attended each other’s events, as did several members of the UK team.

In the second part of the interactive discussion, attendees divided into self-selected groups, and following a brief briefing from the local research team (including community engagement specialists LK, FO, MC, LuK, TC, HM), they were encouraged to discuss potential solutions to the issues raised in the F2P study in their communities. Each group was given ample time to discuss, and each grouping shared their discussion points with the wider group - in their own language, which was then verbally and simultaneously translated to English for the team, who took notes. During the visualization and dissemination events, the research team commissioned a local media company in each location (Blantyre/Nairobi) to photograph and film the informal settlement community events (Figure 2). With the attendees’ collective verbal consent, these were later disseminated through social media and other media. Readers can see a short video clip that shows this community engagement, with the consent of those who attended in Mukuru (see link: https://doi.org/10.6084/m9.figshare.29046011).

In this way, the visualization and interactive discussion of the study results fully engaged the community and provided multiple conversation points, which helped clarify, validate, and contextualize the findings. The direct engagement of researchers with community members in informal settlements around their localized research findings fostered deep conversations and strengthened community members’ capacity to understand the issues of solid fuel use, HAP, and health impacts, thereby serving as an awareness-raising session. These events have now informed the design of successive proposals to funders for new research. The events were particularly successful with young people in the informal settlements, who were very vocal about their community’s needs and have remained engaged since then as informal local groups.

Moreover, the informal settlement communities in Mukuru and Ndirande went beyond simply discussing the findings presented to them and proposed potential solutions that would fit their own contexts. We present them in Table 1, based on notes taken by the research team. The informal settlement community members emphasized the importance of differentiating between what was within their own power to change and what required action by other actors or authorities.

**Table 1:** Potential solutions to the issues suggested by Mukuru and Ndirande community members.

Future actions suggested by Mukuru and Ndirande community members to mitigate HAP and health harms from the use of solid fuels for cooking	Who is responsible?
Empowering the community with knowledge on causes and impacts of solid fuels to their health and well-being as well how to minimize exposure and dangers.	Community members, community leaders & chiefs, NGOs, community health workers, churches, schools, district health authorities and other district stakeholders
Empower ‘champions’ or change agents in the community who can share knowledge with others (peer education).	Researchers and community members with community leaders
Foster entrepreneurial skills and help create job opportunities in the sector to help people to switch to cleaner fuel options (LPG, electricity).	Government (city and national) & community leader
Build better homes with proper ventilation.	Government (city and national) landlords and community leaders
Provide access to affordable clean fuels and create lasting solutions. Subsidize LPG gas prices; reduce the price of electricity and improve supply.	National Government

It is key to note from Table 1 that, although addressing the health impacts of HAP and solid fuels were high on the agenda for informal settlements residents, they also expressed other needs for more empowerment, skill building, and other structural changes (e.g., better ventilated houses, cheaper prices for cleaner fuels). Since these events, community chiefs and community members have supported our Research team in developing further bids by providing support letters and, in one case, becoming co-applicants on a proposal.

### 3.2 ‘Cascading’ the community engagement around research dissemination with policymakers and other key stakeholders

To maximize the impact of the first community discussion events, we conducted a second wave of engagement events with another community of practice or interest: policymakers and other key stakeholders in Nairobi and Blantyre, a few days later. At earlier informal settlements community events in Mukuru and Ndirande, we had asked and obtained verbal collective consent to share the points raised at the local level with a group of policymakers and other key stakeholders in Blantyre and Nairobi.

**Figure 3:**
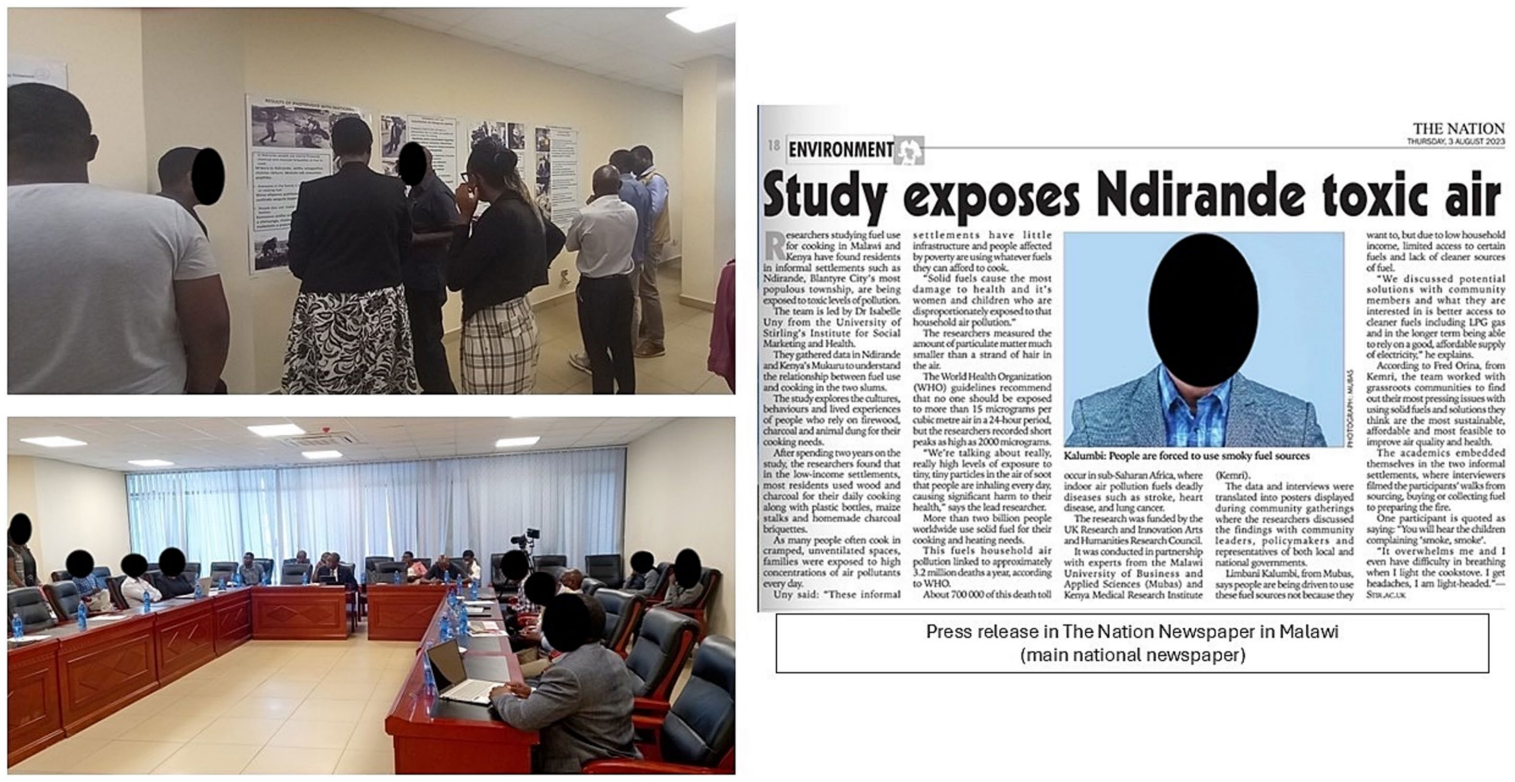
Interactive dissemination event with policymakers in Blantyre (Malawi) and press release.

We thus cascaded our community engagement over two further days of interactive dissemination (again with lunch provided). Those who attended from the policymakers and other stakeholders community were 20 national and district government officials, civil society organizations, non-governmental actors (e.g., NGOs working on clean fuels and clean cookstoves, and various environmental agencies), traditional authority leaders, and media organizations. At these events in Kenya and Malawi, the research team formally presented the study findings and invited the policymakers and key stakeholders to undertake a ‘gallery walk’ around the same poster exhibition (Figure 3) that we had shown the communities. By cascading community engagement, we provided key national and district stakeholders with a ‘right of response’ to issues raised by the informal settlement residents regarding our research findings. Therefore, we created a space for them to share their perspectives as well. The research team took notes of the discussion points, with collective verbal consent from those in attendance, and took some photos to document the proceedings. Local media outlets (press/TV) were present at the engagement events in Nairobi and Blantyre, and press releases were issued in Kenya, Malawi, and the UK, which were picked up by the national and local press. We show photos from the event in Figure 3, along with an associated press release.

Similar to the discussion with the informal settlement community representatives, the policymaker community offered their perspectives on the points raised and solutions mooted by local residents on the ground. They offered short-, medium-, and long-term solutions that they perceived as feasible for reducing HAP exposure and improving public health in informal settlements. We present those in Table 2. Policymakers and other stakeholders recommended actions that were within their own spheres of influence—e.g., national and district governments—and identified potential barriers to action. This community of policymakers and key stakeholders was, unsurprisingly, concerned with the availability and affordability of cleaner fuels, with rolling out clean cooking technologies, and with promoting electricity and LPG as better options to protect public health. They also noted that some of the needs of informal settlement community members require significant resources and investments, which are currently lacking and may only be achievable in the long term.

**Table 2:** Potential actions and solutions raised by policymakers and key stakeholders in Blantyre and Nairobi.

Solutions proposed	Potential barriers
Future actions recommended by policymakers and key stakeholders in Blantyre (Malawi) and Nairobi (Kenya) and to reduce HAP harms from the use of solid fuels for cooking	
Malawi	
Short to medium term: Improve charcoal briquette production at the local level to use fewer polluting components and ensure affordability	Affordability and acceptability of using ‘cleaner’ components to manufacture briquettes
Medium term: Roll out fuel efficient and improved cookstoves (ICS) programs (provide technologies and education) using the infrastructure and enabling environments of the National Cookstove Steering Groups and associations	Cost and uptake of ICS technologies
Longer term: Promote the use and uptake of LPG; Government should subsidize the cost of LPG gas and equipment, drop associated tax and improve distribution. Ensure availability, distribution and affordability of electricity in informal settlements; develop mini-hydro power plants and solar farms.	LPG prices are not set by National Governments (so there is less control) Electricity grids are insufficient, and prices remain high. This requires a substantial investment
Longer term: Explore waste-to-energy solutions (e.g., biogas) Increase research on acceptability and feasibility of suggested innovations.	Lack of available funding
Key Points: The switch to clean fuel in informal settlements will take time and short-term, mid-term and long-term solutions are required. The Government needs to continue to effectively implement existing energy strategies and policies such as the Malawi National Charcoal Strategy 2017–2027; the Government of Malawi (GoM) Renewable Energy Strategy; the Malawi National Energy Policy.	
Kenya	
Short term: Lift tax on LPG and LPG technologies (e.g., LPG VAT was reduced to 8% since 2022); Subsidize clean fuel use	Energy prices and LPG prices are set outside Kenya Affordability to end users in informal settlements
Short term: promote the production of briquettes made of cleaner components	Acceptability of products and manufacturing at scale
Short term: Educate residents on the use of clean fuels for health improvements	Contextually appropriate, co-developed education campaigns; lack of collaboration between Government and NGOs working in that sector
Medium-term: Work with landlords to build affordable houses with proper kitchens and ventilation as well as electric hook ups	Cost of building houses
Longer term: Build micro-energy plants (solar, biogas etc) in the informal settlements around schools, health centers and other community spaces	Government resources
Longer term: Conduct feasibility studies on any suggested solutions before full scale implementation	Funding for research; adequate reporting Not enough community engagement in research and action
Key Points: The switch to clean fuel will take time and short-term, mid-term, long term solutions are required. Governments need to continue to effectively implement existing strategies and policies such as the 2018 The National Energy Policy; the 2020 Kenya National Energy Efficiency and Conservation Strategy and the Kenya Energy Transition & Investment Plan (2023).	

Through the process, this other community (policymakers/other key stakeholders) not only engaged with the research but also increased their capacity to relate to the issues and concerns raised by the informal settlement’s residents. For the research team, the event strengthened relationships with policymakers and other key stakeholders and enhanced the team’s ability to transform ideas into project proposals and tangible actions. As we developed further proposals for funding in the year following, we received letters of support from the ministry representatives, as well as the city and the district councils.

Direct engagement with diverse communities, such as informal settlement residents, policymakers and other stakeholders around our findings, strengthened the capacity of the research team to understand the issues from diverse points of view and contexts. It offered a sense of direction for future research and action (for instance, both the informal settlements communities and the policymakers agreed on the need for more education and awareness about the harms of solid fuel use and HAP). Moreover, these experiences enhanced our interdisciplinary, multi-country research team’s capability to conduct interactive dissemination, a learning that team members have since applied in other projects within their respective institutions and presented at conferences (75). These community-engaged dissemination events have cemented relationships of trust and offered a platform to build on for future HAP research, with the same communities on the ground, to test the feasibility and acceptability of contextually appropriate interventions in informal settlement environments in Malawi and Kenya.

## 4 Discussion

### 4.1 What do we know about take the out dissemination in global public health research?

Across global public health research, community engagement is widely presented as central to research quality, ethical practice, innovation uptake, and sustainability (37, 39, 48, 76, 77). Reviews emphasize that engagement should span the research cycle, from agenda-setting and design through implementation, evaluation, and dissemination, and should explicitly include marginalized or disempowered groups (37). However, research dissemination is still frequently treated as a final, largely one-way process (from researchers to participants or other stakeholders), and is often underreported in published accounts. This creates a gap in practical guidance on how to conduct dissemination in a more interactive, inclusive, and participatory manner rather than as a purely passive or transmissive exercise.

A growing body of work reframes dissemination as a dialog: a reciprocal process in which participants and communities can question, interpret, and actively use findings, rather than simply receive them (45, 46). This matters ethically, as communities have a right to know and respond to the research they contribute to (52); dialog can build trust and improve the relevance and usability of findings (41, 44, 46, 49). Such dissemination approaches are also increasingly linked to decolonial and equity-oriented commitments in global public health, including efforts to recognize and value local knowledge and to reduce extractive research practices (47, 48, 60). Nevertheless, detailed reporting of how interactive dissemination is implemented, particularly across multiple community types (e.g., affected communities and policymakers), remains limited.

In HAP research in Africa, dissemination and direct engagement with communities most affected around findings are minimally reported (58). A small number of studies describe more interactive approaches, including community forums and participatory interpretation (19); workshops and performances (17); community exhibitions that use visual methods to stimulate dialog (32); radio programs and informal gatherings (33); and feedback via embedded fieldworker (59). While these examples are useful, they remain exceptions.

### 4.2 What does this case study add?

Our unique case study describes in detail a process of interactive, cascaded dissemination, from the community to decision makers. It first took the findings to those most affected by HAP in their own voices and pictures. We built on relationships developed during 2 years of participatory fieldwork in those informal settlements and presented the findings to the community members as a poster exhibition, with community members images from the photovoice, graphs from their walking interviews and narratives in their language. This enabled a two-way, supported discussion across diverse literacy levels, allowing participants to question, validate, and contextualize findings from their community in real time; it also enabled them to propose contextually appropriate solutions. Secondly, we took the same exhibition and the discussion points from the community event and built them into a cascaded dissemination with policymakers and other stakeholders, which allowed them to hear community voices and to have a right of response to those and to the researchers’ findings. This process created a structured pathway for community-derived priorities to be carried forward into policy discussions, helping connect lived experience with decision-making spaces. This is consistent with the call for models of “dissemination as dialog,” which stress trust-building, mutual learning, and iterative exchange rather than one-way communication (45, 46).

This type of dissemination process could feasibly be integrated into frameworks for Integrated Knowledge Translation (IKT) (78–80). This framework, which is mostly Africa-borne (81), is defined as a process in which researchers and knowledge users (e.g., policymakers, practitioners, NGOs) collaborate as equal partners throughout the research process. They co-develop research questions, select methods, interpret findings together and plan how research results will be applied. IKT aims to address the “know-do gap” disconnect by making research more relevant, actionable, and actually used (82). IKT purposefully involves knowledge users who have genuine authority to implement change. We contend that the form of interactive, multi-step dissemination we undertook contributes to the IKT framework approach by bringing the lived experience and potential solutions expressed by those most affected to those with genuine authority over programs, budgets, and regulatory decisions, and therefore the capacity to translate priorities into action.

This responds to a recurring gap in community-engaged research, where community perspectives are generated but not systematically integrated into decision making, by offering a clear mechanism for linking micro-level engagement to macro-level policy processes (44, 55). In this way, the cascading model may help to further bridge the “know-do gap” in global public health, producing research that is more actionable and better understood by those who can act on it, while also surfacing practical constraints and opportunities for implementation. At the same time, strengthening IKT-consistent practice would require clearer feedback loops back to communities (e.g., what actions were taken, what was not feasible, and why), in line with the emerging ethical guidance for dissemination and implementation research (54, 57). Overall, our model advances the field by operationalizing dissemination as a multi-level, dialogic process that not only returns findings to participants but actively embeds their perspectives within the policy discourse.

### 4.3 Recommendations for practice

Drawing together our case study and related literature (45, 46, 55, 57), we recommend that researchers use the principles below, to develop dissemination activities which go beyond the usual, performative dissemination modalities and to engage in a vital two-way dialog with communities and other stakeholders (particularly those who have the power to enact change) around the dissemination of their public health research.

1. **Engagement**: Conceptualize community engagement and inclusion as a continuous loop from project ideation and methodological development to dissemination and research usability. As you co-develop new proposals or co-design new interventions, consider engaging directly with prior research findings as a vital first step in the next research cycle (with different communities, be they affected populations or policymakers)
2. **Networks**: Use all available networks to maximize the impact of your research dissemination events at local and national levels. Consider a variety of outputs to disseminate through different channels: peer-reviewed papers, print and social media, TV and radio, short briefs, and community meetings and exhibitions. This will increase impact, facilitate further learning, and enable the research to reach wider audiences. It may support the translation of research into action.
3. **Accessibility**: Ensure that disseminated findings are accessible and visualized in ways that maximize approachability and interaction with the local communities engaged. Ensure that the language also fits the message. Consider whether results could be presented visually, such as posters, videos, photos, art, songs, music, theater, or other folk media methods (78, 81), which may be better suited to certain contexts. Visual, arts-based and other folk media also work well with policymakers and other communities of interest, as they may make a more direct impact.
4. **Capacity strengthening**: Use the interactive dissemination activities to strengthen capacity at all levels, from researchers to communities, policy makers, civil society organizations, funders and others, to address the issues tackled by your research.
5. **Trust**: Build on the trust and relationships established with stakeholders and communities involved in the research from the beginning, and continue to do so when planning and undertaking your interactive dissemination. Conceptualize the interactive sharing and discussion of your research findings as further trust-building, thereby addressing the historical epistemic injustices still inherent in global public health research (48, 67, 77).
6. **Budgeting**: Ensure a dedicated, sufficient budget is allocated to conduct meaningful community-engaged dissemination at the start of your project. This will lead to more meaningful engagement at all levels and will help ensure inclusive participation. As funding for global public health research becomes increasingly constrained, we also urged funders to allow proposals to allocate resources to this vital part of the research cycle (83).

To make public health research more impactful, interactive community engagement should be intentionally incorporated into the dissemination phase. Too often, dissemination is treated as a one-way process, in which researchers present their findings without inviting participants to reflect on, interpret, and act on the research results within their lived contexts. Through community-engaged dissemination, communities can utilize research findings to address or propose solutions to their local issues. We recommend that public health researchers use these principles and reframe dissemination as a dialogic and participatory process.

### 4.4 Limitations

Although separate meetings ensured openness within each group and helped manage power imbalances, this approach likely reduced opportunities for direct dialog, co-learning, and joint prioritization between community members and policymakers. Future work could test sequenced joint forums or other formats that enable safer multi-stakeholder interaction. While the cascading model strengthened “upward” communication of community-derived priorities to stakeholders with authority to act, we did not formally evaluate downstream outcomes (e.g., policy decisions, implementation actions, or longer-term changes) following dissemination. Finally, interactive dissemination is time- and resource-intensive (translation, facilitation, material, venue and participation support) and may be difficult to deliver at scale without dedicated funding and institutional commitment. In our F2P project, this extensive community-engaged dissemination was made possible by additional funding from the University of Stirling. With yet more resources (staff time and funding), we could have conducted a series of interactive dissemination events in various informal settlements in Kenya and Malawi, thus increasing knowledge exchange and impact. This would have facilitated further community engagement and enhanced the usability and applicability of the research findings.

## 5 Conclusion

Our work shows the importance of involving communities at every stage of public health research, especially when sharing results. We also demonstrate that research participants can propose solutions to challenges identified in the research. Treating dissemination as a collaborative dialog helps researchers better understand the local context, make findings more relevant to stakeholders, and strengthen trust. The principles advance this collaborative process by supporting meaningful community engagement throughout the research process and enabling communities to use findings to promote equitable health outcomes.

## Data Availability

Publicly available datasets were analyzed in this study. This data can be found at: all relevant data are supplied within the paper.
